# The awareness and determinants of diabetic foot ulcer prevention among diabetic patients: Insights from NHANES (2011–2018)

**DOI:** 10.1016/j.pmedr.2023.102433

**Published:** 2023-09-21

**Authors:** Xingkai Wang, Mengying Xu, Lei Meng, Mingzhi Song, Zhuqiang Jia, Lin Zhao, Xin Han, Shouyu Wang, Junwei Zong, Ming Lu

**Affiliations:** a Department of Trauma and Tissue Repair Surgery, Dalian Municipal Central Hospital, Dalian, China; b Department of Orthopaedic Surgery, The First Affiliated Hospital of Dalian Medical University, Dalian, China; c The First Affiliated Hospital of Nanhua Medical University, Hengyang, China; d The First Affiliated Hospital of Dalian Medical University, Dalian, China; e Naqu People's Hospital, Tibet, China; f Department of Quality Management, Dalian Municipal Central Hospital, Dalian, China; g Department of Orthopaedic Surgery, The Second Affiliated Hospital of Dalian Medical University, Dalian, China

**Keywords:** Diabetic foot ulcer, Prevention, Risk factors, NHANES, Hypertension

## Abstract

The prevention of diabetic foot ulcers (DFU) precedes treatment, in that early prevention significantly reduces the incidence of foot ulcers. The main objectives of this study were to examine the current prevalence of proactive foot ulcer examinations among diabetic patients and analyze influencing factors, in order to provide a scientific reference for the prevention of DFU in diabetic patients. The National Health and Nutrition Examination Survey (NHANES) 2011–2018 (n = 1278) data were utilized in this cross-sectional study. The dependent variable was whether patients underwent self-initiated foot ulcer inspections; risk factors that may lead to foot ulcers were included as independent variables. To explore the connection between the patient’s subjective motivation to inspect foot ulcers and risk variables, the weighted logistic regression model was further carried out. Among all risk factors, race, body mass index (BMI) and hypertension were statistically significant between whether patients were examined for foot ulcers or not. In the fully adjusted logistic regression model, only hypertension was positively correlated with diabetic patient-initiated examination for foot ulcers. This study suggests that there is still room for improvement in the knowledge and behavior of diabetic patients to be proactive in preventing DFU. Health care and community workers should conduct targeted training on diabetic foot prevention to reduce and prevent DFU by reinforcing knowledge to build positive attitudes and drive preventive behavior change.

## Highlights

• 80% Self-reported diabetics had foot exams in 8 years; 17% did not, showing awareness needs.
• Ethnicity influenced exams; Non-Hispanic Whites were proactive. Targeted education is crucial.
• High BMI not linked to exams; hypertension in diabetics correlated with foot checks.
• Patient education is key to preventing DFUs. Healthcare must prioritize foot protection and overall health.

## 1 Introduction

Diabetes is a metabolic condition defined by excessive glucose levels, and diabetes or its consequences afflict more than 30 million individuals in the United States alone (Divers et al., 2020). Diabetes is predicted to cost $327 billion per year, with 73% spent on direct healthcare expenditures and the other 27% spent on diabetes-related comorbidities (Economic Costs of Diabetes, 2017).

Diabetic foot ulcer is one of the more serious vascular complications of diabetes, affecting roughly 6.3% of the world's population (Zhang et al., 2017). Diabetic foot ulcer is a foot infection, ulcer and deep tissue destruction caused by abnormal nerves and varying degrees of vasculopathy in the distal lower limbs of diabetic patients (Mariadoss et al., 2022). Diabetic foot ulcers (DFU) is one of the most devastating consequences of diabetes, causing significant pain and financial hardship for individuals. Its most typical clinical symptom is chronic ulceration, which can lead to amputation/toe amputation or even death. It has been claimed that the lifetime risk of getting foot ulcers is as high as 25%, with an annual incidence of 2–7% (Balasubramanian et al., 2020, Kasbekar et al., 2017, Ning et al., 2019).

The mechanisms underlying the development of foot ulcers in diabetic patients are complex and result from a combination of many intrinsic and extrinsic factors. Several major risk factors include: peripheral neuropathy, history of amputation or foot ulceration, foot deformity, peripheral vascular disease, visual impairment, renal disease, poor glycaemic control and smoking (Fesseha et al., 2018, Chow et al., 2016). The Wagner classification is the most widely used international rating system for evaluating the progression of diabetic foot conditions. It was established by Meggitt et al. in 1976 and then modified by Wagner for clinical use in 1981 (Wagner, 1987, Wagner, 1981). With a higher Wagner classification, there is a greater likelihood of amputation and a lower rate of cure and improvement. Grades 0 and 1 on the Wagner classification represent the presence of risk factors and superficial ulcers without signs of infection, respectively, which are high risk states for the development of DFU. The risk is often overlooked by diabetics because there is no obvious clinical manifestation, leading to many diabetics suffering from gangrene. Accordingly, early recognition and active prevention can improve the quality of life of patients and delay the progression of foot ulcers. With the above in mind, in this study we used NHANES data to examine the current prevalence of proactive foot ulcer examinations among diabetic patients and analyze influencing factors. Based on their results and influencing factors, targeted strategies to promote patients' active participation in DFU prevention are proposed, providing a scientific reference base for diabetic patients in DFU prevention.

## 2 Related work

Previous research has played a pivotal role in advancing our understanding of factors influencing the prevalence of diabetic foot ulcers (DFU) among diabetic patients. Investigations into factors such as body mass index (BMI), income levels, and age have shed light on the complexities of DFU development. These studies have elucidated the significance of intrinsic and extrinsic factors that contribute to DFU risks.

In the conventional theory of cognitive thought, there are differences in the perception of various items by age, gender, education level and even poverty status. Notably, the literature has indicated that the incidence of foot ulcers in diabetic patients increases with age, and is more common in males than females (Zhang et al., 2017). Studies had shown that the occurrence of diabetic foot ulcers increased with the amount of smoking (Zhang et al., 2020). The nicotine in tobacco stimulates the release of epinephrine and norepinephrine, causing vasoconstriction and spasm, which results in tissue ischaemia and hypoxia and reduced tissue perfusion making diabetics prone to foot ulcers (Upputuri et al., 2020, Ren et al., 2019). Basit et al. found that poor glycaemic control and high HbA1c stimulated endothelial cell apoptosis, accompanied by the production of large amounts of terminal glycosylation products, leading to luminal narrowing, which was strongly associated with diabetic foot ulcers (Ehlers et al., 2019, Basit et al., 2005). Elevated blood lipids cause large amounts of lipid to invade the vessel wall, thickening the basement membrane and narrowing the lumen of the artery, causing an increase in endogenous clotting factors and predisposing the formation of blood clots, leading to diabetic foot ulcers (Regenhardt et al., 2018). Although there is no clear relationship between walking limitations and the development of DFU, in type 2 diabetics, peripheral neuropathy is associated with fewer steps per day but no significant mobility impairment (van Sloten et al., 2011). Because of the disturbance of calcium and phosphorus metabolism in the body due to renal insufficiency, itching and dryness of the skin are often caused, and scratching is likely to break the skin and bruise it, once combined with infection will lead to diabetic foot. The diabetic foot and diabetic retinopathy are both microvascular complications of diabetes mellitus, and a correlation between the pathogenesis of the two has been demonstrated (Sellman et al., 2018). However, even in the presence of risk factors that clearly result in DFUs, the prevalence continues to increase, suggesting that diabetic patients lack awareness of self-prevention of foot ulcers and have not yet realized the importance of effective assessment in the prevention of DFU.

Efforts have been directed towards elucidating the complex interplay between risk factors and preventive measures. By examining the multifaceted relationship between diabetic patients' demographic characteristics, clinical parameters, and proactive foot examination behavior, researchers have worked towards establishing a comprehensive framework for DFU prevention. As the understanding of DFU has evolved, researchers have sought to integrate these findings into actionable recommendations for healthcare professionals and patients alike. By combining empirical evidence with analytical insights, studies have endeavored to bridge the gap between research outcomes and real-world applications, ultimately contributing to the enhancement of clinical practice and patient care.

## 3 Proposed methodology

In this cross-sectional study, the correlation between risk factors associated with DFU and the proactive foot examination behavior of diabetic patients was investigated. The data for this cross-sectional study were gathered from the NHANES website (https://wwwn.cdc.gov/nchs/nhanes/Default.aspx), which was a nationally representative assessment of nutrition and health conditions in the United States. We analyzed the data from the last 4 cycles (2011–2018). All NHANES studies were conducted in accordance with the Declaration of Helsinki, all participants provided informed consent, and the National Center for Health Statistics (NCHS) Research Ethics Review Board authorized them.

Inclusion and exclusion criteria were next established based on DFU-related risk factors and the presence of diabetes mellitus, and patients who met the criteria were screened for the study. Finally, utilizing a weighted logistic regression model as the decision mechanism, the correlation between risk factors associated with DFU and the proactive foot examination behavior of diabetic patients was explored, providing a scientific reference basis for the prevention of DFU among individuals with diabetes.

## 4 Experimental setup

### 4.1 Population

**Fig. 1:**
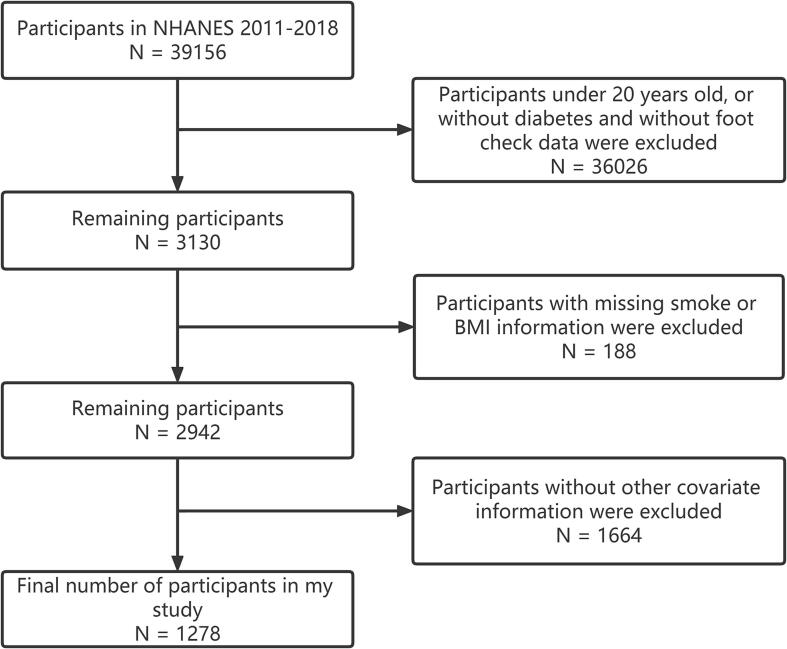
Participant enrollment and analysis flowchart.

A total of 39,156 subjects were enrolled in 4 consecutive NHANES survey cycles covering the periods 2011–2018. There were 36,026 participants who were under 20 years old or did not have diabetes and did not have foot examination data in this study. Then, the participants who had no data on BMI, smoking behavior and other covariates were also eliminated, resulting in the final research population of 1,278. Fig. 1 depicts the recruiting procedure.

### 4.2 Study variable

The dependent variable was assessed from the question, “How often {do you check your feet/does SP check (his/her) feet} for sores or irritations? Include times when checked by a family member or friend, but do not include times when checked by a doctor or other health professional.” This was then dichotomized as checking feet: yes (greater than1 times) or no (0 times).

**Fig. 2:**
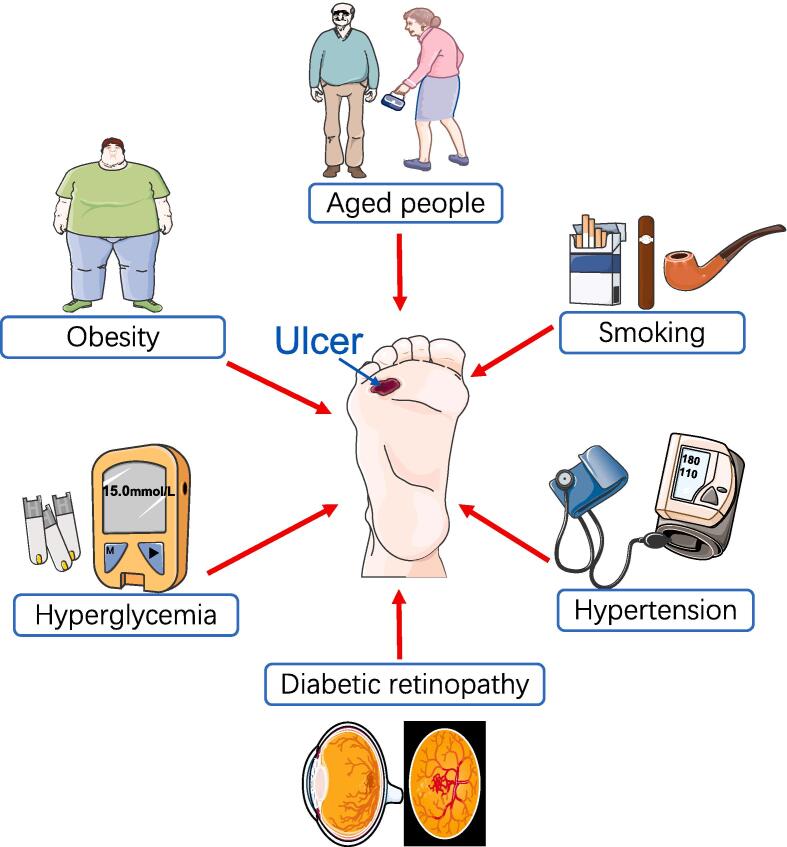
Key risk factors associated with diabetic foot ulcers.

The independent variables were chosen with reference to the extensive literature on selected risk factors for diabetic foot ulcers **(**Fig. 2**)**. Demographic characteristics were obtained from the NHANES Demographics Data and included age, sex, race and education level. Age was grouped as 20–49, 50–79, ≥80 years old; sex was classified as male and female; the ethnicity variable contains Non-Hispanic White, Non-Hispanic Black, Mexican American, Other/multiracial and Other Hispanic; the education level is categorized as Any College, High school and Less than High school. BMI (kg/m2) is divided into three levels, with < 25.0 considered healthy weight, 25.0–29.9 regarded as obese and ≥ 30.0 as overweight. Smoking status was categorized as never, former, and current. Glycated hemoglobin (HbA1C) is classified into three grades, ranging from < 5.75 as normal, 5.75–6.5 as re-diabetes and otherwise as abnormal. Other covariates included dyslipidemia, hypertension, walking limitations, diabetic retinopathy, renal failure, stroke and coronary heart disease, the details of which can be obtained at https://www.cdc.gov/nchs/nhanes/.

### 4.3 Statistical analyses

Due to the consideration that the NHANES survey oversampled participants, we integrated the sample weights created in the NHANES analysis. Descriptive statistics use weighted frequencies for categorical variables and medians and 95% confidence intervals for continuous variables to describe subjects. The logistic regression was performed to assess the association between risk factors and foot check after adjusting for potential confounding factors. Three models were built: an unadjusted model, a minimally adjusted model (adjusted for age, sex, and race), and a fully adjusted model (age, sex, race, education level, BMI, smoking, glycohemoglobin, dyslipidemia, hypertension, walking limitations, diabetic retinopathy, renal failure, stroke and coronary heart disease). A p-value < 0.05 (two-sided) was statistically significant.

## 5 Results

The demographics and characteristics of participants with the foot ulcers examined in this study are presented in Table 1. From 2011 to 2018, there were 1,278 people over the age of 20 with diabetes and data on the presence of foot ulcers examined. 83% of participants reported actively checking their feet to observe for ulcers. The average age of participants was 61.0 years old with a similar number of males and females. The majority of those who underwent foot examinations in this study had an education level at college, yet after weighted correction, there was no statistical significance. The same applies to smoking status, with the more diabetic people who never smoked paying attention to foot examinations, but it was not statistically significant. Ethnic differences in the proportion of diabetic subjects who had their feet examined were significant, with Non-Hispanic White being the group that focused more on foot examination compared to other ethnic groups. The level of importance placed on foot examinations varies among the healthy weight, obese and overweight populations, with obese having the most foot examinations, followed by overweight. In addition, apart from risk factors other than demographic characteristics, only hypertension demonstrated statistical differences between subjects.

**Table 1:** Demographics and characteristics of study participants from NHANES 2011–2018.

**Characteristic**	**N**^1^	**Overall**, N = 1278 (100%)^2^	**Foot Check**, N = 1059 (83%)^2^	**No Foot Check**, N = 219 (17%)^2^	**P Value**^3^
**Age (years)**	1,278	61.0 (53.0, 70.0)	61.0 (52.0, 70.0)	61.4 (53.1, 71.0)	0.6
**Age group**	1,278				0.8
*20*–*49 years*		200 (18)	164 (18)	36 (17)	
*50*–*79 years*		909 (71)	753 (70)	156 (73)	
*80 + years*		169 (12)	142 (12)	27 (10)	
**Sex**	1,278				0.2
*female*		610 (49)	508 (50)	102 (42)	
*male*		668 (51)	551 (50)	117 (58)	
**Race**	1,278				**0.003**
*Non-Hispanic White*		408 (60)	340 (60)	68 (57)	
*Non-Hispanic Black*		321 (14%)	289 (16%)	32 (7.5)	
*Mexican American*		217 (9.5)	182 (9.5)	35 (9.8)	
*Other/multiracial*		184 (9.9)	127 (8.3)	57 (18)	
*Other Hispanic*		148 (6.5)	121 (6.3)	27 (7.4)	
**Education level**	1,278				0.3
*Any College*		591 (53)	497 (53)	94 (52)	
*High school*		279 (26)	233 (26)	46 (22)	
*Less than High school*		408 (22)	329 (21)	79 (26)	
**BMI**	1,278				**0.014**
*Healthy Weight*		176 (11)	132 (11)	44 (14)	
*Obese*		714 (60)	624 (62)	90 (48)	
*Overweight*		388 (29)	303 (27)	85 (38)	
**Smoking**	1,278				0.11
*Current smoker*		192 (14)	157 (14)	35 (12)	
*Former smoker*		439 (36)	363 (34)	76 (45)	
*Never smoker*		647 (50)	539 (51)	108 (43)	
**HbA1C**	1,278				0.077
*Abnormal*		880 (69)	751 (71)	129 (61)	
*Normal*		111 (7.9)	89 (7.4)	22 (9.9)	
*Pre-Diabetes*		287 (23)	219 (22)	68 (29)	
**Dyslipidemia**	1,278				0.7
*Dyslipidemia*		722 (59)	620 (60)	102 (58)	
*Normal*		556 (41)	439 (40)	117 (42)	
**Hypertension**	1,278				**0.008**
*Hypertensive*		892 (70)	766 (72)	126 (60)	
*Normal*		386 (30)	293 (28)	93 (40)	
**Walking Limitations**	1,278				0.6
*Normal*		962 (78)	785 (78)	177 (80)	
*Walking limitations*		316 (22)	274 (22)	42 (20)	
**Retinopathy**	1,278				0.3
*Normal*		1,038 (84)	847 (83)	191 (87)	
*Retinopathy*		240 (16)	212 (17)	28 (13)	
**Renal Failure**	1,278				>0.9
*Normal*		1,133 (90)	934 (90)	199 (89)	
*Renal Failure*		145 (10)	125 (10)	20 (11)	
**Stroke**	1,278				0.083
*Normal*		1,169 (92)	964 (91)	205 (95)	
*Stroke*		109 (8.4)	95 (9.2)	14 (4.6)	
**Coronary Heart Disease**	1,278				0.6
*Coronary Heart Disease*		133 (12)	119 (12)	14 (9.5)	
*Normal*		1,145 (88)	940 (88)	205 (90)	
**Notes:**^1^N not Missing (unweighted)					
^2^Median (IQR) for continuous; n (%) for categorical					
^3^Wilcoxon rank-sum test for complex survey samples; chi-squared test with Rao & Scott's second-order correction					
**Abbreviations:** BMI, Body mass index; HbA1C, Glycated hemoglobin.					

Based on these results, the logistic regression models between BMI, hypertension and whether the subject initiated a foot examination was analyzed separately **(**Table 2**)**. We found a significant positive association between obese and foot examination and a significant negative association between overweight and foot examination in the unadjusted model (model 1). However, after adjustment for covariates, no significant associations were found for these models (models 2 and 3). Hypertension was positively associated with active foot examination in three models with statistically significant: model 1 (OR = 1.74, 95% CI: 1.15 – 2.62, p-value = 0.007), model 2 (OR = 1.67, 95% CI: 1.10 – 2.55, p-value = 0.014), model 3 (OR = 1.68, 95% CI: 110 – 2.58, p-value = 0.014).

**Table 2:** Association Between BMI, Hypertension, and Foot Examination in NHANES 2011–2018.

	**Model1**			**Model2**			**Model3**		
**Characteristic**	**OR**^1^	**95% CI**^1^	**p-value**	**OR**^1^	**95% CI**^1^	**p-value**	**OR**^1^	**95% CI**^1^	**p-value**
**BMI**			**0.017**			0.11			0.4
Healthy Weight	Reference	Reference		Reference	Reference		Reference	Reference	
Obese	1.67	0.94, 2.96		1.39	0.74, 2.58		1.20	0.60, 2.42	
Overweight	0.94	0.52, 1.69		0.86	0.47, 1.58		0.86	0.44, 1.68	
**Hypertension**			**0.007**			**0.014**			**0.014**
Normal	Reference	Reference		Reference	Reference		Reference	Reference	
Hypertensive	1.74	1.15, 2.62		1.67	1.10, 2.55		1.68	1.10, 2.58	
**Notes:** Model 1: no covariates were used for adjustment. Model 2: age, sex, race were used for adjustment. Model 3: age, sex, race, education level, BMI, smoking, HbA1C, dyslipidemia, hypertension, walking limitations, diabetic retinopathy, renal failure, stroke and coronary heart disease were used for adjustment.									
**Abbreviations:**^1^OR, Odds Ratio; CI, Confidence Interval									

## 6 Discussion

The findings of this study showed that approximately 80% of patients with a self-reported diagnosis of diabetes would have voluntarily examined their feet in the past eight years. Despite the relatively high proportion of patients with diabetes who initiate foot examinations, the proportion of patients without an active foot examination in the last 8 years is still quite high (17%). The study was conducted using NHANES data, a population-based survey, which represents the approximately 3.9 million people with diabetes with no active examination of their feet during the survey period. It is a worrying statistic showing the lack of awareness of foot ulcers among diabetics, leading to a multi-fold increase in the likelihood of foot ulcers being one of the most common clinical causes. Health care and community workers should provide targeted and individualized health education to patients with diabetes. Early identification and timely effective intervention of risk factors for DFU is essential for the management of DFU.

In this study, we introduced age, sex, race, poverty status, education level, BMI, smoking, glycohemoglobin, dyslipidemia, hypertension, walking limitations, diabetic retinopathy, renal failure, stroke and coronary heart disease as risk factors associated with patient-initiated foot examinations, and calculated the proportion of each factor in the different populations, as well as analyzing the correlation with active foot examinations in diabetic patients. However, these risk factors (age, smoking, HbA1c, etc.) associated with the development of DFU in this study were not significantly different between diabetic patients who actively examined their feet and those who did not, suggesting that diabetic patients lack awareness of self-prevention of foot ulcers and have not yet realized the importance of effective assessment in the prevention of DFU.

A significant correlation was found between the prevalence of disease and ethnic variability, particularly in diabetes, so that the occurrence of diabetic foot may also differ between ethnic groups (Zhang et al., 2017). The corresponding results in this study indicated that the importance of foot examinations varied by ethnicity, with the Non-Hispanic White population being the most important group for foot examinations, which may also account for the lower limb amputations in this group compared to Black/African Americans (Miller et al., 2022). Among the diabetic population, BMI above normal levels is associated with an increased chance of lower limb complications, which was also reflected in this study (Gray et al., 2015, Shen et al., 2012). However, there was no positive association between obesity and active foot examination after correction for covariates in the regression analysis. Elevated systolic blood pressure is an independent risk factor for the development of diabetic foot ulcers. A prospective study also found that systolic blood pressure was significantly higher in the diabetic foot ulcer group than in the non-diabetic foot ulcer group (Ikem et al., 2010). Prolonged hypertension leads to weakened arterial wall elasticity, reduced compliance, increased intima-media thickness and endothelial cell damage, as well as reduced production of nitric oxide by endothelial cells or its bioavailability, thus accelerating the formation of atherosclerosis (Magarinos et al., 2013, Kostov and Halacheva, 2018). The destruction of endothelial cells impairs vascular self-regulation and reduces blood supply to the foot, resulting in tissue ischaemia and hypoxia, leading to the development of diabetic foot ulcers. In addition, the regression analysis of this study also revealed a positive correlation in that diabetic patients with hypertension were more likely to initiate foot examinations.

This study revealed that foot ulcers are predominantly concentrated among diabetic patients. The study analysis revealed that diabetic patients with high blood pressure were more proactive in checking for foot ulcers, possibly because blood pressure measurements were more easily done on a regular basis. The patients are actively checking their feet for ulcers whenever their blood pressure increases. Nevertheless, in summary, elevated blood pressure is only one possible cause of DFU, and the convenience of checking blood pressure leads patients to focus only on blood pressure and thus ignore other causes. Therefore, the specialist medical staff should provide patients and their families with more forms and richer contents of education related to foot protection and strengthen health education on prevention of diabetic foot. In clinical practice, it is advisable for healthcare providers to pay special attention to hypertensive patients when treating individuals with diabetes and encourage them to actively engage in foot observation. Furthermore, in clinical settings, we emphasize that healthcare professionals need to take a comprehensive approach, advocating not only for proactive and vigilant foot observation but also addressing risk factors associated with foot ulcers, such as diabetes control, blood pressure management, and foot health. These health education measures enable patients to detect early prediabetic foot lesions, strengthen self-behavioral management and prevent the occurrence of ulcers.

Additionally, this study invokes population data in the NHANES database of 2011–2018, which suffers from a short time period and an inadequate population base, and the awareness of DFU prevention among the population has now changed over time. In subsequent studies, we will extend the time period and increase the population base, and combine the clinical patient follow-up data to provide a scientific reference for the prevention of DFU.

## 7 Conclusion

We analyzed 1278 diabetic patients from the 2011–2018 cross-sectional study using NHANES data to uncover risk factors associated with promoting subjective examination of foot ulcers in diabetic patients. The results indicated that among the many risk factors, only hypertension was positively correlated with active examination of foot ulcers in diabetic patients. This study suggests that there is room for improvement in the knowledge and behavior of diabetic patients to proactively prevent DFU and that awareness of the potential risk factors for DFU needs to be increased so as to essentially reduce the incidence of DFU. Health care and community workers should conduct targeted training on diabetic foot prevention to reduce and prevent DFU by reinforcing knowledge to build positive attitudes and drive preventive behavior change.

## CRediT authorship contribution statement

**Xingkai Wang:** Conceptualization, Formal analysis, Validation, Writing – original draft. **Mengying Xu:** Data curation, Investigation, Methodology. **Lei Meng:** Software, Visualization, Writing – original draft. **Mingzhi Song:** Methodology, Software. **Zhuqiang Jia:** Writing – original draft. **Lin Zhao:** Software. **Xin Han:** Visualization. **Ming Lu:** Project administration, Resources, Supervision, Writing – review & editing. **Junwei Zong:** Project administration, Resources, Supervision, Writing – review & editing. **Shouyu Wang:** Conceptualization, Funding acquisition, Project administration, Resources, Supervision, Writing – review & editing.

## Declaration of Competing Interest

The authors declare that they have no known competing financial interests or personal relationships that could have appeared to influence the work reported in this paper.

## Data Availability

Data will be made available on request.
